# Intraoperative HIFU Ablation of the Pancreas Using a Toroidal Transducer in a Porcine Model. The First Step towards a Clinical Treatment of Locally Advanced Pancreatic Cancer

**DOI:** 10.3390/cancers13246381

**Published:** 2021-12-20

**Authors:** Celia Cilleros, Aurélien Dupré, Yao Chen, Jeremy Vincenot, Michel Rivoire, David Melodelima

**Affiliations:** 1 LabTAU, INSERM, Centre Léon Bérard, Université Lyon 1, Univ Lyon, F-69003 Lyon, France; celia.cilleros@inserm.fr (C.C.); aurelien.dupre@lyon.unicancer.fr (A.D.); yao.chen@lyon.unicancer.fr (Y.C.); michel.rivoire@lyon.unicancer.fr (M.R.); 2 EDAP TMS, 4 Rue du Dauphiné, F-69120 Vaulx-en-Velin, France; jvincenot@edap-tms.com

**Keywords:** ultrasound, pancreas, locally advanced, tumor, Doppler guidance

## Abstract

**Simple Summary:**

Regardless of treatment, the overall 5-year survival rate for patients with pancreatic adenocarcinoma is less than 5%. The aim of this preclinical study was to evaluate the feasibility of intraoperative focused ultrasound ablation of the pancreas under Doppler guidance to treat the pancreatic parenchyma and tissues surrounding the superior mesenteric vessels in vivo in a pig model. Large and homogeneous destruction of the pancreatic parenchyma and tissues around the peripancreatic artery without spasm or occlusion by focused ultrasound during an open procedure was feasible without organ penetration. Ultrasound guidance allows for the objective evaluation of the actual treated region. This method could aid in the treatment of locally advanced pancreatic adenocarcinoma that is inaccessible by other known therapeutic methods. The device presented herein is simple to use, reliable and adaptable to local conditions. Such a treatment may also be used in conjunction with resection.

**Abstract:**

Apart from palliative chemotherapy, no other therapy has been proven effective for the treatment of locally advanced pancreatic tumors. In this study, an intraoperative high-intensity focused ultrasound (HIFU) device was tested in vivo to demonstrate the feasibility of treating the pancreatic parenchyma and tissues surrounding the superior mesenteric vessels prior to clinical translation of this technique. Twenty pigs were included and treated using a HIFU device equipped with a toroidal transducer and an integrated ultrasound imaging probe. Treatments were performed with energy escalation (from 30 kJ to 52 kJ). All treatments resulted in visible (macroscopically and in ultrasound images) homogeneous thermal damage, which was confirmed by histology. The dimensions of thermal lesions measured in ultrasound images and those measured macroscopically were correlated (r = 0.82, *p* < 0.05). No arterial spasms or occlusion were observed at the lowest energy setting. Temporary spasm of the peripancreatic artery was observed when using an energy setting greater than 30 kJ. The possibility of treating the pancreas and tissues around mesenteric vessels without vascular thrombosis holds great promise for the treatment of locally advanced pancreatic cancers. If clinically successful, chemotherapy followed by HIFU treatment could rapidly become a novel treatment option for locally advanced pancreatic cancer.

## 1. Introduction

Pancreatic adenocarcinoma is among the most aggressive of all cancers. Regardless of treatment, the overall 5-year survival rate for this disease is less than 5% and has shown only minimal improvement during the past few decades [1,2,3]. The majority of patients are treated with palliative intent due to either metastatic or locally advanced disease. When surgical resection is feasible, the 5-year survival rate is approximately 20%. However, surgery is possible in only 15–20% of patients [4,5]. Considering the high rate of unresectability and the poor results of surgery alone in patients with pancreatic carcinoma, many treatment efforts incorporating chemotherapy, radiotherapy, or both have been made to improve the 5-year survival of these patients. As most patients (80–90%) are diagnosed with advanced (30–40%) or metastatic (50–60%) pancreatic carcinoma, the development of improved systemic treatment options has been a top priority over the past two decades. Patients with no metastatic disease but an unresectable tumor are defined as having locally advanced pancreatic adenocarcinoma (LAPA). This group represents 30 to 40% of patients. Treatment remains highly controversial, as it confers an average overall survival of only 9 to 14 months regardless of the treatment strategy. In this subgroup of patients, chemotherapy remains the standard of care. The combination of radiation therapy and chemotherapy is not recommended, as it has not demonstrated any survival advantages [6,7]. Unfortunately, most of these patients have a very limited chance of undergoing surgery even after chemotherapy [8]. Given the high incidence of locally advanced pancreatic cancer and the low probability of downstaging with conventional treatment (chemotherapy and radiotherapy), there has been a growing interest in the use of new local ablative therapies, such as radiofrequency ablation (RFA) and irreversible electroporation (IRE), for multimodal treatment of the disease. These ablative techniques are applied to ultimately induce irreversible cellular damage to the tumor, leading to cell death via either apoptosis or coagulative necrosis [9,10,11].

Among these new treatment options, high-intensity focused ultrasound (HIFU) is a recently developed technology that uses therapeutic ultrasound; focused beams pass harmlessly through superficial tissue and produce sufficiently strong heating in the focal zone to cause irreversible tissue necrosis in only a few seconds. Most HIFU treatments are performed with an extracorporeal approach that has been largely reported as a palliative option to treat patients with LAPA, with pain relief observed in 78–100% of the patients [12,13,14,15,16]. Mild complications including abdominal pain, nausea and vomiting, skin burn, and subcutaneous fat sclerosis usually occur in 3–20%. Some case series have reported adverse events such as pancreatic pseudocysts and pancreatitis in 10% of patients. The median survival time of patients with LAPA after treatment with HIFU either alone or in combination with concurrent chemotherapy has been reported to range from 10.0 to 12.6 months [12]. Although the extracorporeal use of HIFU for treating pancreatic carcinoma has been proven safe and feasible, to date, it is limited to palliative treatments with objective pain reduction, as no clear evidence of survival benefits has been reported. The use of extracorporeal HIFU treatment for pancreatic cancer patients has been limited due to several factors mainly related to the deep location of the pancreas in the abdomen and its poor acoustic access. The surrounding structures (stomach, colon, liver, and kidney) present very different acoustic properties, leading to differential attenuation and phase aberration that can change the shape of the focal zone and the acoustic intensity at the focus [17]. LAPA treatment has also been affected by difficulties in obtaining clear percutaneous imaging of the tumor shape and margins.

Given the inconvenience and disadvantages of extracorporeal HIFU, we developed an intraoperative HIFU probe that was initially designed for the treatment of colorectal liver metastasis and reported encouraging results [18,19]. The principal focus lies in the possibility of treating the parenchyma independently from perfusion [20] with high control of the treatment margins [21] and accurate visualization of the tumor and of HIFU ablation [18,19,22,23]. The intraoperative HIFU probe designed for the treatment of liver tumors has shown potential applicability for pancreatic tumors [24]. Treating LAPA with HIFU intraoperatively can also help to confirm that the tumor is not resectable (major vascular invasion precluding resection) and that liver or peritoneal metastatic spread has not occurred. In the case of technical unresectability, HIFU ablation could be a suitable alternative. In contrast, in the case of metastatic spread not visualized on preoperative imaging, ablation should not be performed. Particular attention must be given to the superior mesenteric blood vessels when treating LAPA since irreversible mesenteric occlusion can cause mesenteric ischemia, which is a life-threatening condition [25,26,27]. Vascular complications caused by extracorporeal HIFU treatment have been reported, including secondary occlusion of the superior mesenteric artery and portal vein thrombosis [28]. Therefore, the aim of this preclinical study was to evaluate the feasibility of intraoperative HIFU thermal ablation of the pancreas with energy escalation under Doppler guidance to treat the pancreatic parenchyma and tissues surrounding the superior mesenteric vessels in vivo in a pig model prior to clinical translation of this technique.

## 2. Materials and Methods

### 2.1. Animal Model

Local institutional review board approval was obtained before conducting experiments at the Institute of Experimental Surgery of the Leon Berard Cancer Center. The animal experiments described in this paper conformed to the requirements of the French National Institute of Health and Medical Research Office of Animal Experimentation and were performed in accordance with the legal requirements of the European directives on Animal Experimentation.

The porcine pancreas presents some differences from the human pancreas. Instead of joining the common bile duct, the main duct drains into the duodenum directly [29]. In addition, the porcine pancreas is divided into the duodenal lobe, the connecting lobe and the splenic lobe. In terms of their anatomical arrangement, these three parts look like the head, uncinate process, and the body and tail of the pancreas in humans, respectively [30]. In a similar way to that in humans, the head of the porcine pancreas (duodenal lobe) is connected to the duodenum, the body (splenic lobe) follows the curved shape of the stomach and the tail end of the splenic lobe extends to the left kidney. Importantly, the artery along the portal vein is not the mesenteric artery, as in humans, but the hepatic artery. However, the diameter of this hepatic artery (5 mm) is similar to the diameter of the mesenteric artery in humans. Despite these differences, the similarities in terms of the anatomical dimensions and structure between the human and porcine pancreas make the pig the predominant model for preclinical studies on the treatment of pancreatic carcinoma with HIFU [24,31,32,33,34,35,36]. Therefore, in this study, the feasibility of selective ablation of the porcine pancreas and tissues around the peripancreatic hepatic artery using an intraoperative toroidal HIFU transducer was investigated under Doppler guidance in 20 healthy, female landrace pigs 12 to 14 weeks old and weighing between 25 and 30 kg.

### 2.2. HIFU Equipment

**Figure 1:**
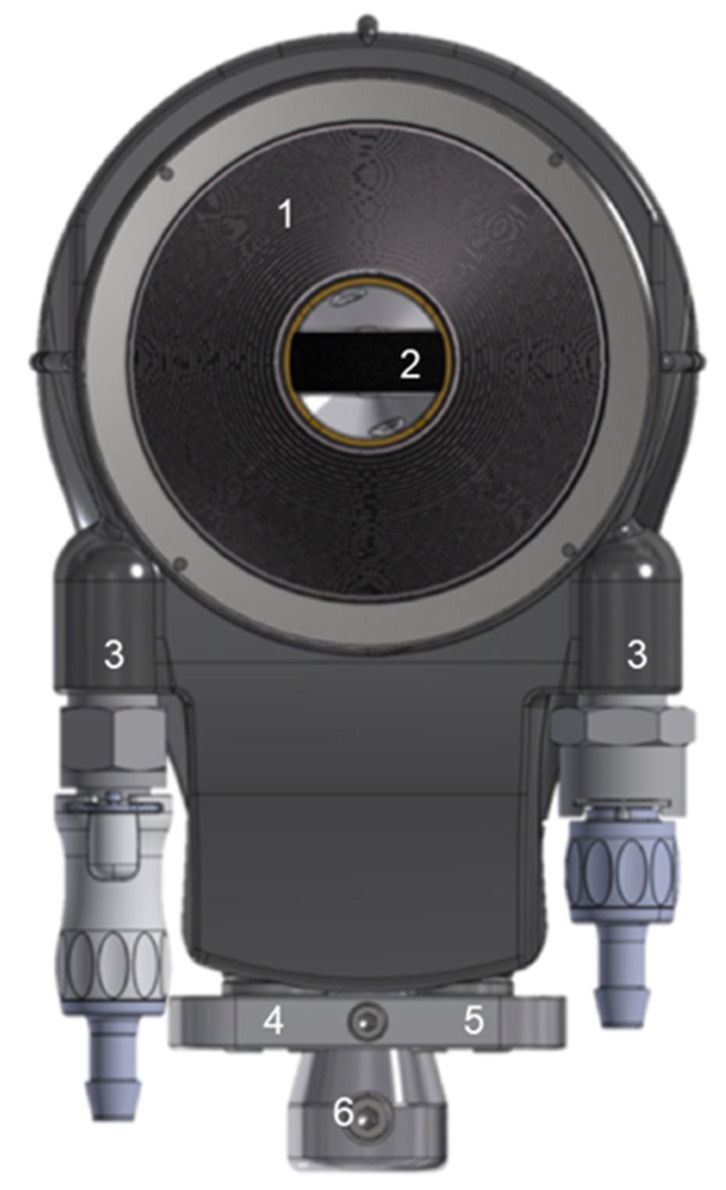
Schematic of the HIFU intraoperative device. 1: HIFU transducer divided into 32 concentric rings. 2: Ultrasound imaging probe. 3: Connectors for the cooling circuit. 4: Connectors for the coaxial cables of the HIFU emitters. 5: Connectors for the cables of the ultrasound imaging probe. 6: Connector for the mechanical arm.

The toroidal HIFU transducer was divided into 32 concentric rings with equal surface areas (78 mm^2^) and its diameter was 68 mm. The central frequency was 2.5 MHz. A 7-MHz ultrasound imaging probe composed of 192 elements (X12C3e, Vermon, Tours, France) was placed in the center of the HIFU transducer (Figure 1). The HIFU acoustic axis was aligned with the ultrasound imaging plane. A sterile polyurethane coating (CIV-Flex Transducer cover, Ref 610-004, CIVCO, Kalona, IA, USA) was used to cover the HIFU device. This coating attenuated the ultrasound pressure by 2% at 2.5 MHz. Degassed sterile water (sterile water, Ref 882-1315, Baxter, Guyancourt, France) was used to fill the coating and to provide a coupling fluid between the HIFU transducer and pancreatic tissues. The water was maintained at 7 °C. A peristaltic pump drove the water with a continuous flow of 0.5 L/min in a closed circuit to cool the HIFU transducer during sonication.

In the focal plane, the focal zone created by a toroidal transducer was a ring measuring 30 mm in diameter. Moreover, an overlapping area was created between this focal ring and the transducer. This overlapping area increases the size of the lesion that could be created [37]. An ablation rate of 10.5 cc/min [18] could be performed without resorting to mechanical scanning, even in highly perfused organs such as the liver [20]. Focusing on a large area involves fewer mechanical effects (boiling and cavitation) and promotes thermal destruction, which results in a clear evaluation of the treated zone [18,24]. The HIFU transducer was controlled by an electronic system (MSonic^®^, EDAP-TMS, Vaulx-en-Velin, France) composed of two 16-channel power amplifiers with a master-slave mode (Pige-Electronique, Bourg-lès-Valence, France), an ultrasound scanner (BK3000 scanner, BK 5000 soft version, BK medical, Herlev, Denmark), a peristaltic pump (PMD24 OEM, Watson Marlow Fluid Technology Group, Falmouth, UK) and a computer. The maximal electrical power deliverable per channel was 20 W. Each channel was programmable in phases with a resolution of 1° for electronic focusing of the HIFU beam. A directional coupler was placed inside each channel of the power amplifiers to measure the direct and reflected electrical power. The position of the HIFU focal region was indicated on the sonograms, making it possible to place the location of the ablation precisely in tissues [38]. Ablations were observed using the ultrasound imaging probe integrated in the HIFU transducer and also by using a 14 MHz ultrasound imaging probe (Model I14C5I, BK Medical, Herlev, Denmark) to obtain high-resolution images.

### 2.3. Treatment Procedure

The animals arrived on-site 7 days before the beginning of the experiments and were fasted 24 h before the treatment. Premedication was performed using an intramuscular injection of a mix of ketamine (10–12 mg/kg) and azaperone (4–5 mg/kg). A 20-gauge catheter was placed in the auricular vein, and 7 mL of 1% propofol was injected immediately before intubation. An assisted ventilation system (ABT 4300, Kontron Instruments) supplied oxygenation at a rate of 7.2 l.min^−1^ and a frequency of 12 cycles.min^−1^. Anesthesia was maintained during the procedure with continuous infusion of 1% propofol (15 mL/h). Peroperative analgesia was administered by continuous perfusion of sufentanil (3 mL/h). Blood oxygen saturation was monitored by an optical captor and maintained at 100%. Hydration was provided by isotonic perfusion of a 9% physiological saline solution. Animals were positioned in the dorsal decubitus position and classical surgical asepsis was performed. A 25 cm median laparotomy was performed from the xyphoid process. The tail of the pancreas was freed from its attachments and folded in the targeted area to more closely mimic the thickness of the human pancreas (typically 15 mm).

**Figure 2:**
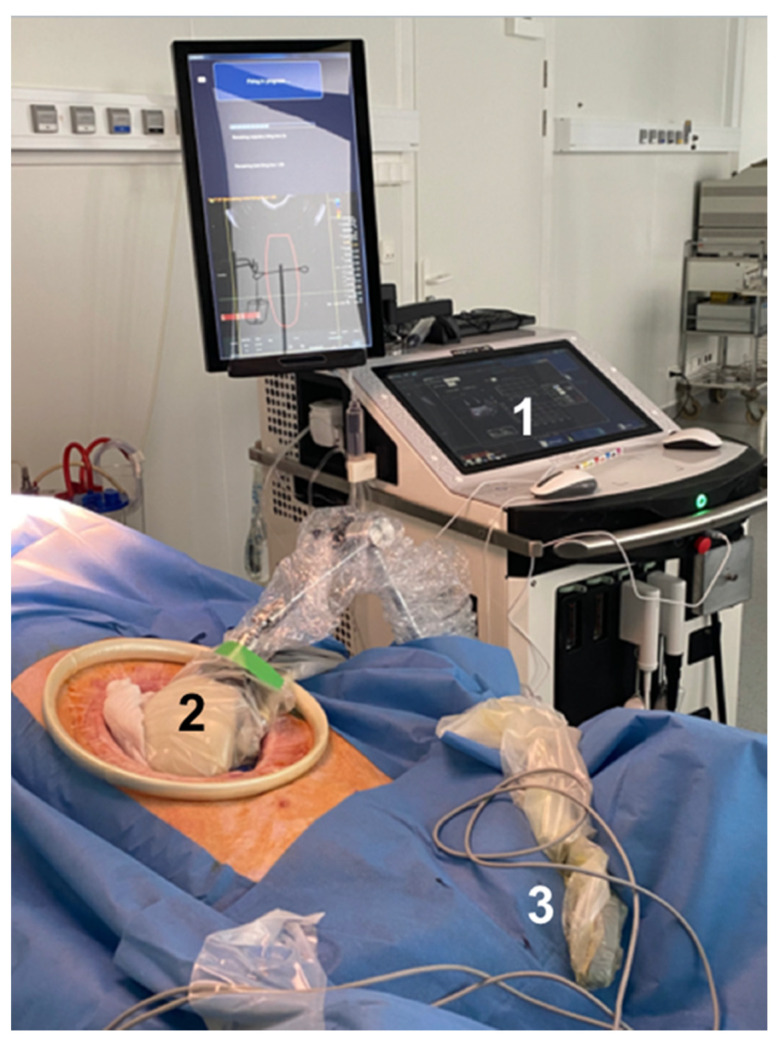
Experimental setup during in vivo treatment. 1: Electronic system used to control the HIFU transducer. 2: Intra-operative HIFU device. 3: Additional ultrasound imaging probe working at 14 MHz.

The HIFU device was held by a mechanical arm and placed in acoustic contact with the pancreas (Figure 2). The pancreas and peripancreatic vessels were clearly visible in ultrasound images thanks to the open procedure. Moreover, pads can be used to protect organs in the vicinity of the pancreas to eliminate the risk of secondary lesions. The region to be treated was located using the integrated ultrasound imaging probe. HIFU sonication procedures were performed on healthy pancreatic parenchyma and tissues surrounding the peripancreatic artery. Doppler images were acquired just before each HIFU sonication procedure and then during sonication. Doppler imaging was also performed just after HIFU treatment to visualize the effects on the peripancreatic artery.

Prior to the animal experiments, treatment parameters were estimated from numerical simulations and adjusted in vitro using a perfused model [38]. An initial in vivo study (not described in this paper) that uses the same animal model was then conducted using three pigs to optimize the treatment parameters. In all cases, the total exposure time was 900 s with a duty cycle of 40% HIFU exposure (10 s) and 60% Doppler color imaging (15 s). All HIFU sonication procedures were performed by electronically setting the depth of the ring-shaped focus between 65 and 90 mm and its radius between 0 (spherical focalization) and 15 mm (toroidal focalization). HIFU sonication was performed without moving the probe. Animals were treated using underrated acoustic power set to 83 W (*n* = 8), 110 W (*n* = 4), 124 W (*n* = 4) or 143 W (*n* = 4), corresponding to energies of 30, 40, 45, and 52 kJ, respectively. These sonication parameters were defined to create lesions approximately 20 mm in diameter. Up to three HIFU sonication procedures were permitted if normal blood flow in the peripancreatic hepatic artery was observed after each sonication. The 14 MHz ultrasound imaging probe was also used to observe the lesions with higher resolution. The longest and shortest axes of the treated area visible in sonograms were measured after each treatment. Possible effects on adjacent organs, burns in particular, were carefully investigated throughout the entire abdominal cavity. The laparotomy was closed in two planes, and a dressing was placed to protect the wound.

After treatment, the animals were returned to their pens, and their general behavior was observed twice daily for signs of anorexia, distress, or fever. Postsurgical analgesia was provided using a fentanyl patch (100 µg/h) and an intramuscular injection of Profenid (100 mg) that could be readministered if needed. Fifteen days after HIFU treatment, the animals were anesthetized, and laparotomy was performed using the same procedure described above. Additional ultrasound images of the HIFU lesions were acquired using the 14 MHz ultrasound imaging probe. The animals were euthanized under general anesthesia immediately after treatment by pentobarbital injection (20 mL). The entire peritoneal cavity, including the pancreas and adjacent organs, was examined during autopsy. The pancreas was removed and sliced to visually inspect the effects of ultrasound. Each HIFU lesion was sectioned transversally and then sliced into 3 mm thick samples to determine whether the thermal damage was homogeneous. Representative samples were then fixed in a 10% solution of formaldehyde. After 48 h, the samples were transferred to phosphate-buffered saline and then dehydrated using increasing concentrations of alcohol, treated with intermedium, and embedded in paraffin. The embedded samples were sliced and stained using hematoxylin and eosin. Microscopic analyses were performed by a laboratory that specialized in veterinary histological analysis (Vet Diagnostics, Lyon, France).

### 2.4. Data Analysis

An even surface containing a reference scale was used to take photographs of each sample. The software ImageJ, version 1.52a (http://imagej.nih.gov/ij, accessed on 9 September 2020) was used to analyze images of the lesions. On each sample, the shortest and longest axes of the treated area, visible as a white necrotic core, were measured. The examiner who measured the dimensions of the lesions on the sonograms was different from the examiner who measured the dimensions via gross pathology. All measured data are expressed as the mean ± standard deviation (minimum value–maximum value).

## 3. Results

### 3.1. Overview

In total, 20 HIFU ablation procedures were performed in the pancreas and tissues surrounding the peripancreatic hepatic artery in 20 pigs using this intraoperative device. The sonication results are summarized in Table 1 and Table 2. All sonication procedures resulted in visible (both macroscopically and using conventional ultrasound imaging) and palpable damage at the target site. Nine treatments induced coagulative necrosis, which was confirmed by histology. Eleven treatments induced cystic lesions with localized necrosis. The occurrence of cystic lesions was independent of the treatment parameters (Table 2) but due to the direct effect of heat on the main pancreatic duct.

### 3.2. Clinical Tolerance

No deaths occurred before autopsy. The animals tolerated the surgical and HIFU procedures well and without hemodynamic disturbances. There were no visible lesions in the surrounding organs. The clinical course was uneventful for all animals. No cases of severe pancreatitis were observed.

**Table 1:** Sonication results leading to an artery spasm or not.

Pig N°	Group	Emitted Energy (J)	Number of Sonications	Artery Spasm	Number of Sonications before the Artery Spasm
1	1	30	3	No	N/A
2			3	No	N/A
3			3	No	N/A
4			3	No	N/A
5			3	No	N/A
6			3	No	N/A
7			1	No	N/A
8			2	No	N/A
9	2	40	3	No	N/A
10			3	No	N/A
11			3	Yes	3
12			3	Yes	3
13	3	45	2	Yes	1
14			3	Yes	1
15			1	Yes	1
16			3	Yes	1
17	4	52	2	Yes	1
18			2	Yes	1
19			2	Yes	1
20			2	Yes	1

**Table 2:** Dimensions of the lesions measured macroscopically. * Ablations could not be precisely measured because of fragmentation of pancreatic parenchyma.

Pig N°	Group	Emitted Energy (kJ)	Treated Zone	Long Axis (mm)	Shot Axis (mm)
1	1	30	Necrotic	18.2	18.9
2			Cystic	60.3	49.8
3			Necrotic	18.2	12.4
4			Cystic	31.8	25.5
5			Cystic	66.1	50.3
6			Cystic	74.9	36.6
7			Cystic	37.9	30.3
8			Cystic	41.5	34.0
9	2	40	Necrotic	N/A *	N/A *
10			Necrotic	N/A *	N/A *
11			Cystic	51.1	46.9
12			Cystic	46.8	18.7
13	3	45	Necrotic	27.5	25.4
14			Necrotic	23.7	18.3
15			Cystic	43.7	15.2
16			Cystic	31.6	19.6
17	4	52	Necrotic	33.8	18.6
18			Necrotic	35.8	23.9
19			Necrotic	31.9	18.5
20			Cystic	55.2	21.8

### 3.3. Analysis of HIFU Treatment

**Figure 3:**
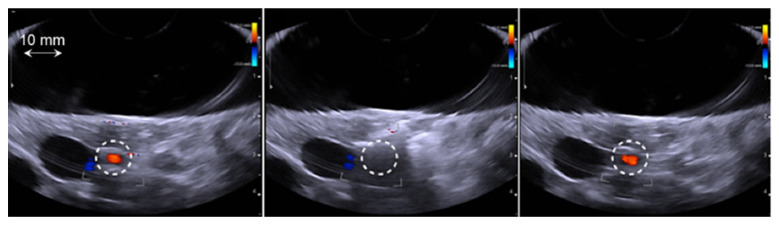
Typical example in group 4 of a temporary spasm. From left to right: Color Doppler imaging of the targeted artery (white circle) just before the HIFU treatment, just after the HIFU sonication and 12 min after the sonication. Normal blood flow was recovered on average 12 min after the temporary spasm in all cases.

The exposure time was constant for all sonication procedures (900 s with a duty cycle of 40% HIFU exposure and 60% Doppler color imaging). The effect of the emitted energy (acoustic power) on the targeted artery was clearly observed (Table 2). No arterial spasms were observed for treatments performed using an emitted acoustic energy of 30 kJ (corresponding to an emitted acoustic power of 83 W), even when the treatment was repeated three times. In this group, Doppler color imaging showed normal blood flow after sonication in all cases. In group 2, which was treated using an emitted acoustic energy of 40 kJ (corresponding to an emitted acoustic power of 110 W), temporary arterial spasm was observed on Doppler imaging in two animals during the third sonication. No blood flow was observed in the treated artery immediately after sonication in these two cases. Normal blood flow was observed on Doppler imaging 12 min after the treatment (Figure 3).

Systematic temporary arterial spasm was observed in groups 3 and 4, which were treated with acoustic energies of 45 kJ and 52 kJ, respectively (corresponding to emitted acoustic powers of 124 W and 143 W, respectively). In these two groups, all spasms occurred during the first sonication. On average, arterial spasm was observed 51 ± 18 s (min: 29 s–max: 71 s) before the end of sonication. Normal blood flow reappeared in the treated artery at an average of 12.6 ± 6.2 min (min: 7 min–max: 22 min) after treatment. Interestingly, it was possible to perform two additional sonication procedures without additional arterial spasm in all cases.

**Figure 4:**
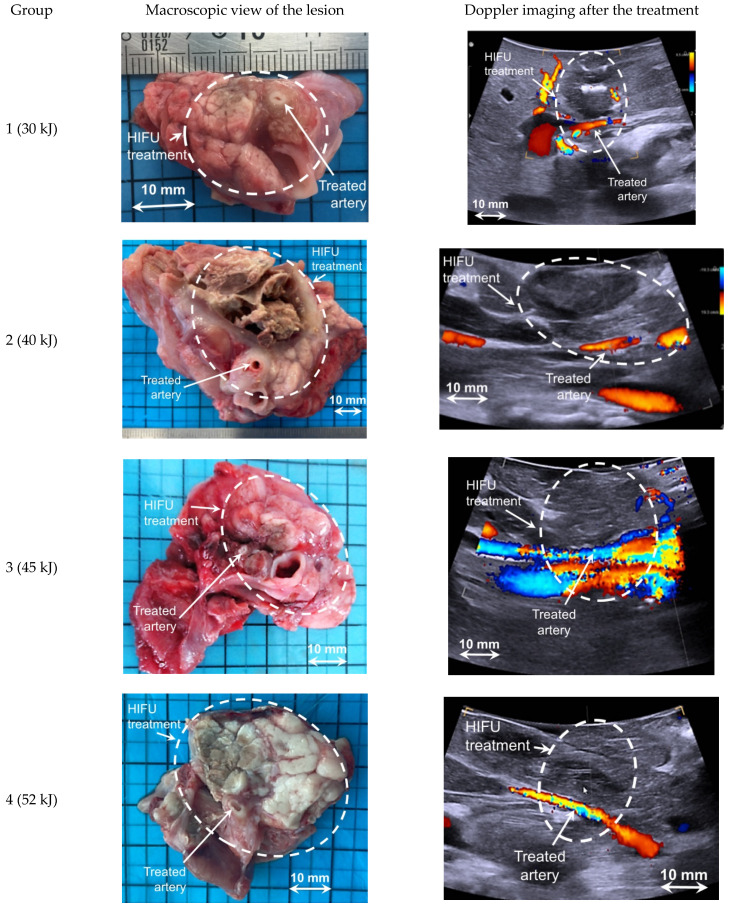
Typical examples for each group of HIFU ablations observed macroscopically and on ultrasound imaging 14 days after treatment.

All treatments induced a lesion that was palpable and easily visible on ultrasound images and macroscopically appeared as a color change in the treated tissue (Figure 4). Treated tissues were harder than untreated pancreatic parenchyma and appeared as a hypoechoic region in the ultrasound images (Figure 4). The lesion dimensions were completely different for cystic and necrotic lesions (Table 2), but a high correlation was found between the dimensions measured on ultrasound images and the dimensions measured macroscopically in all cases (r = 0.82, *p* < 0.05).

**Figure 5:**
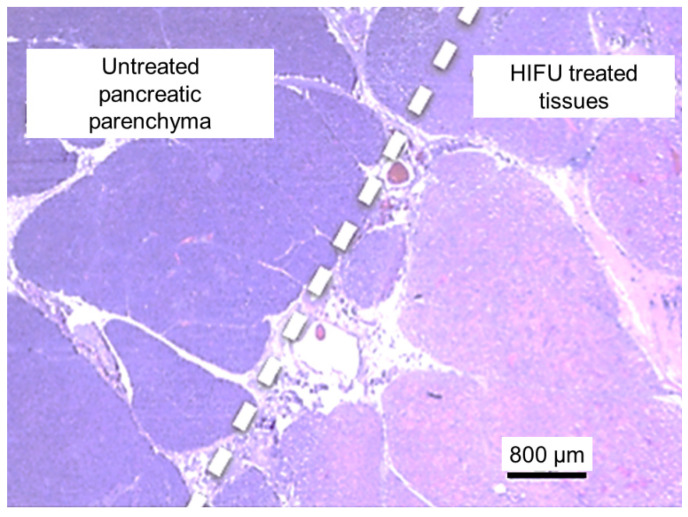
Histological examination of the treated pancreatic parenchyma using hematoxylin and eosin stain. The transition between untreated and treated tissues is typically about 100 µm.

**Figure 6:**
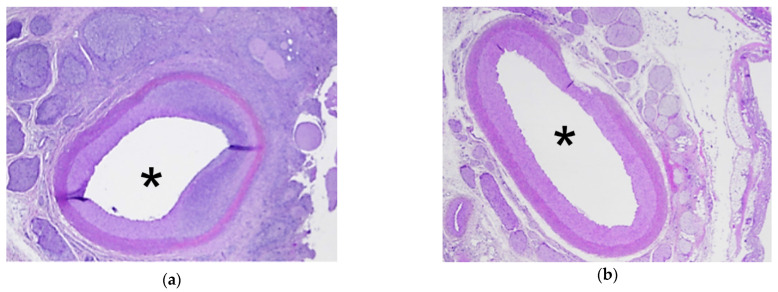

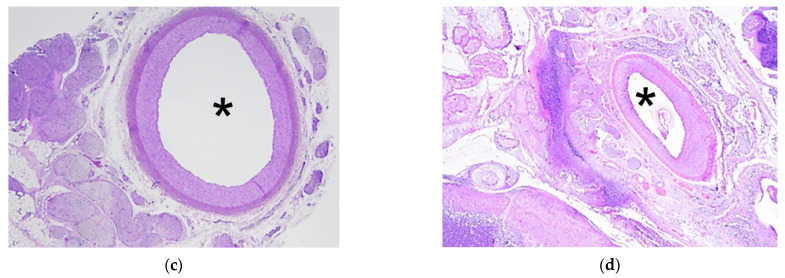
Histological examination of the treated arteries (location indicated by *) for group 1–4 (**a**–**d**). Hematoxylin and eosin stain; magnification ×100.

**Figure 7:**
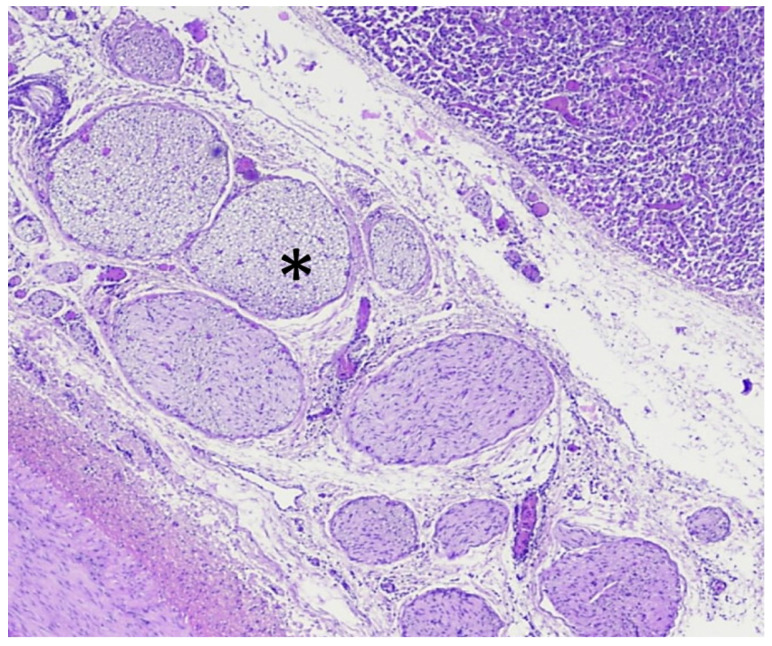
Histological examination of a typical example of the treated plexus nerve (location indicated by *) around the treated arteries. Hematoxylin and eosin stain; magnification ×200.

Histological analyses confirmed that all treatments were homogeneous without any vessel occlusion. Histologically, HIFU ablation corresponded to coagulative necrosis, with a clear delimitation between treated and untreated pancreatic parenchyma (Figure 5). Arterial lesions were characterized by necrosis of the arterial wall, loss of endothelium, condensation of muscle fiber nuclei, disruption of elastic fibers, and necrosis of the outer elastic lamina and adventitia (Figure 6). Necrosis of the peri-arterial adipose tissue was also observed with accumulation of fibrin, small acute hemorrhages, and some neutrophils. Nerve bundles in the periphery of the artery were degenerated in groups 2–4, characterized by vacuolation of the nerve sheaths. These nerve lesions could explain the arterial spasms observed, leading to temporary stenosis. Moreover, these nerve lesions were also responsible for the loss of arterial vasoconstriction capacity, explaining how an artery experiencing an initial spasm could be treated several times thereafter without blood flow being further affected. Arterial necrosis was observed in the lesions in group 4, which was treated with an emitted energy of 52 kJ (Figure 6d). Histological analyses also indicated that plexus nerves around the peripancreatic artery were treated by HIFU in all cases with fibrous tissues observed fifteen days after the procedure (Figure 7).

## 4. Discussion

This study was designed to confirm the feasibility, safety, and efficacy of the intraoperative HIFU ablation of pancreatic parenchyma surrounding the superior mesenteric vessels using the same HIFU device used in humans for the treatment of liver metastases [18]. Treatments with energy escalation under Doppler guidance allowed for the determination of sonication parameters to create a safe thermal ablation procedure at least 18 mm in size in all directions without occlusion of the peripancreatic hepatic artery. HIFU treatments were performed in 15 min. This study also identified the treatment parameters that can induce temporary arterial occlusion. Once a temporary arterial spasm has been generated, sonication can be performed without creating any additional spasm of the artery. This is due to the lesioning of nerve bundles in the periphery of the artery, and no secondary thromboses due to the lesioning of the endothelium of the arteries were observed for up to 14 days. Moreover, we previously demonstrated that HIFU thermal ablation in the pancreas was well tolerated even when pseudocysts were created at the treated site [24]. With a delivered energy of 30 kJ, eight reproducible HIFU thermal ablations were generated in the pancreas and around the peripancreatic hepatic artery without any complications or spasm of the artery observed, and normal blood flow after treatment and homogeneous destruction were achieved. It was also possible to repeat the procedure up to three times in six animals, without any complications observed.

The pancreatic parenchyma is very fragile, and its partial thermal destruction carries the theoretical risk of severe pancreatitis, which needs to be assessed further. The pig pancreas is similar to the human pancreas and enables the preclinical evaluation of this risk, as shown by a preliminary study from our group [24]. In this study, all HIFU thermal ablation procedures were well tolerated. The consequences of HIFU ablation were either pseudocysts or localized necrotic abscesses at the treatment site. The occurrence of pseudocysts could be explained by the direct effect of heat on the main pancreatic duct, with functionality remaining in the distal pancreas. These complications have already been shown to be asymptomatic [24].

The pancreas is an especially difficult organ to image using ultrasound imaging, even intraoperatively. However, the ultrasound imaging probe integrated into the HIFU device confirmed the possibility of visualizing tissue changes after treatment because the dimensions of the observed speckled changes were highly correlated with the treated area in all cases. These results are in agreement with those previously obtained for liver ablation by HIFU [18,39]. This is important because ultrasound imaging provides guidance and allows for the eventual juxtaposition of several HIFU lesions when treating larger tumors [23,40,41].

The prognosis of LAPA is very poor, and few patients have resectable disease. Downstaging was observed in only 15% to 30% of patients treated with chemotherapy and radiotherapy [42,43]. This low rate can be improved with the development of new local therapies, such as HIFU, to increase the number of patients who can be treated with curative intent by becoming candidates for surgery or other treatments. For example, encouraging results regarding the use of intraoperative RFA have been published recently [44,45,46], which highlights its potential for the local destruction of pancreatic tumors. The need for alternative treatments is also demonstrated by the recent development of irreversible electroporation [47,48] for the management of LAPA.

Treatment of pancreatic tumors using the extracorporeal HIFU approach has also been suggested [16,36,49,50,51]. However, the surrounding digestive tract organs may be easily harmed by HIFU making the targeting difficult. Moreover, current HIFU treatments require mechanical scanning for treating LAPA which is extremely difficult to perform with precision percutaneously in a deep-seated abdominal organ [16,50,51]. Therefore, long treatment time are required and guiding the treatment using percutaneous ultrasound imaging is also difficult [50]. The intraoperative approach described herein allows for precise targeting of the region of interest and short treatment times. In surgical oncology contraindications are found intraoperatively in 10% to 20% of patients planned for resection [52]. For example, the discovery of metastatic disease (mainly liver metastases and peritoneal carcinomatosis) clearly precludes local treatment and illustrates the need for an intra-operative treatment. For these reasons the HIFU device used in this study was developed for intraoperative used as the one we developed for liver tumors [18,53]. The size of the ablation can be adapted to constrain related to the organ using electronic focalization [54].

The main limitation of this study is the absence of pancreatic tumors in the treated animals. We targeted healthy pancreatic tissue and healthy peripancreatic hepatic artery because there is no pancreatic tumor model available for pigs.

## 5. Conclusions

In conclusion, destruction of the pancreatic parenchyma and tissues around the peripancreatic hepatic artery without spasm or occlusion of the artery by HIFU during an open procedure is feasible over a short time period and without organ penetration. Ultrasound guidance allows for the objective evaluation of the actual treated region. This method could aid in the treatment of LAPA that is inaccessible by other known therapeutic methods. The device presented herein for pancreatic parenchyma is simple to use, reliable and adaptable to local conditions. Such a treatment may have a role in treating LAPA and may also be used in conjunction with resection. A translational study in humans is warranted to confirm these encouraging results.

## Data Availability

The data presented in this study are available on request from the corresponding author. The data are not publicly available due to confidentiality agreements.

## References

[1] Ryan D.P., Hong T.S., Bardeesy N. (2014). Pancreatic adenocarcinoma. N. Engl. J. Med..

[2] Ishido K., Hakamada K., Kimura N., Miura T., Wakiya T. (2021). Essential updates 2018/2019: Current topics in the surgical treatment of pancreatic ductal adenocarcinoma. Ann. Gastroenterol. Surg..

[3] Wei K., Hackert T. (2021). Surgical Treatment of Pancreatic Ductal Adenocarcinoma. Cancers.

[4] van Veldhuisen E., Jenniskens S.F.M., van den Boezem P.B., Futterer J.J., de Wilt J.H.W. (2019). Locally Advanced Pancreatic Cancer: Work-Up, Staging, and Local Intervention Strategies. Cancers.

[5] Paiella S., de Pastena M., Faustini F., Landoni L., Pollini T., Bonamini D., Giuliani T., Bassi C., Esposito A., Tuveri M. (2019). Central pancreatectomy for benign or low-grade malignant pancreatic lesions—A single-center retrospective analysis of 116 cases. Eur. J. Surg. Oncol..

[6] Auclin E., Marthey L., Abdallah R., Mas L., Francois E., Saint A., Cunha A.S., Vienot A., Lecomte T., Hautefeuille V. (2021). Role of FOLFIRINOX and chemoradiotherapy in locally advanced and borderline resectable pancreatic adenocarcinoma: Update of the AGEO cohort. Br. J. Cancer.

[7] Garnier J., Ewald J., Marchese U., Gilabert M., Moureau-Zabotto L., Giovannini M., Poizat F., Delpero J.R., Turrini O. (2020). Borderline or locally advanced pancreatic adenocarcinoma: A single center experience on the FOLFIRINOX induction regimen. Eur. J. Surg. Oncol..

[8] Garnier J., Ewald J., Marchese U., Gilabert M., Launay S., Moureau-Zabotto L., Poizat F., Giovannini M., Delpero J.R., Turrini O. (2020). Outcomes of patients with initially locally advanced pancreatic adenocarcinoma who did not benefit from resection: A prospective cohort study. BMC Cancer.

[9] Philips P., Hays D., Martin R.C. (2013). Irreversible electroporation ablation (IRE) of unresectable soft tissue tumors: Learning curve evaluation in the first 150 patients treated. PLoS ONE.

[10] Girelli R., Frigerio I., Salvia R., Barbi E., Martini P.T., Bassi C. (2010). Feasibility and safety of radiofrequency ablation for locally advanced pancreatic cancer. Br. J. Cancer.

[11] Paiella S., de Pastena M., D’Onofrio M., Crino S.F., Pan T.L., de Robertis R., Elio G., Martone E., Bassi C., Salvia R. (2018). Palliative therapy in pancreatic cancer-interventional treatment with radiofrequency ablation/irreversible electroporation. Transl. Gastroenterol. Hepatol..

[12] Dababou S., Marrocchio C., Rosenberg J., Bitton R., Pauly K.B., Napoli A., Hwang J.H., Ghanouni P. (2017). A meta-analysis of palliative treatment of pancreatic cancer with high intensity focused ultrasound. J. Ther. Ultrasound.

[13] Marinova M., Rauch M., Mucke M., Rolke R., Gonzalez-Carmona M.A., Henseler J., Cuhls H., Radbruch L., Strassburg C.P., Zhang L. (2016). High-intensity focused ultrasound (HIFU) for pancreatic carcinoma: Evaluation of feasibility, reduction of tumour volume and pain intensity. Eur. Radiol..

[14] Strunk H.M., Lutzow C., Henseler J., Mucke M., Rauch M., Marx C., Schild H.H., Marinova M. (2018). Mesenteric Vessel Patency Following HIFU Therapy in Patients with Locally Invasive Pancreatic Cancer. Ultraschall Med..

[15] Marinova M., Strunk H.M., Rauch M., Henseler J., Clarens T., Brux L., Dolscheid-Pommerich R., Conrad R., Cuhls H., Radbruch L. (2017). High-intensity focused ultrasound (HIFU) for tumor pain relief in inoperable pancreatic cancer: Evaluation with the pain sensation scale (SES). Schmerz.

[16] Strunk H.M., Henseler J., Rauch M., Mucke M., Kukuk G., Cuhls H., Radbruch L., Zhang L., Schild H.H., Marinova M. (2016). Clinical Use of High-Intensity Focused Ultrasound (HIFU) for Tumor and Pain Reduction in Advanced Pancreatic Cancer. Rofo.

[17] Tanter M., Pernot M., Aubry J.F., Montaldo G., Marquet F., Fink M. (2007). Compensating for bone interfaces and respiratory motion in high-intensity focused ultrasound. Int. J. Hyperth..

[18] Dupre A., Melodelima D., Perol D., Chen Y., Vincenot J., Chapelon J.Y., Rivoire M. (2015). First clinical experience of intra-operative high intensity focused ultrasound in patients with colorectal liver metastases: A phase I-IIa study. PLoS ONE.

[19] Dupre A., Melodelima D., Perol D., Chen Y., Vincenot J., Chapelon J.Y., Rivoire M. (2019). Evaluation of the Feasibility, Safety, and Accuracy of an Intraoperative High-intensity Focused Ultrasound Device for Treating Liver Metastases. J. Vis. Exp..

[20] Melodelima D., N’Djin W.A., Favre-Cabrera J., Parmentier H., Rivoire M., Chapelon Y.J. (2009). Thermal ablation produced using a surgical toroidal high-intensity focused ultrasound device is independent from hepatic inflow occlusion. Phys. Med. Biol..

[21] N’Djin W.A., Melodelima D., Parmentier H., Rivoire M., Chapelon J.Y. (2010). In vivo preclinical evaluation of the accuracy of toroidal-shaped HIFU treatments using a tumor-mimic model. Phys. Med. Biol..

[22] Melodelima D., N’Djin W.A., Parmentier H., Chesnais S., Rivoire M., Chapelon J.Y. (2009). Thermal ablation by high-intensity-focused ultrasound using a toroid transducer increases the coagulated volume. Results of animal experiments. Ultrasound Med. Biol..

[23] Parmentier H., Melodelima D., N’Djin A., Chesnais S., Chapelon J.Y., Rivoire M. (2009). High-intensity focused ultrasound ablation for the treatment of colorectal liver metastases during an open procedure: Study on the pig. Ann. Surg..

[24] Dupre A., Melodelima D., Pflieger H., Chen Y., Vincenot J., Kocot A., Langonnet S., Rivoire M. (2017). Thermal Ablation of the Pancreas With Intraoperative High-Intensity Focused Ultrasound: Safety and Efficacy in a Porcine Model. Pancreas.

[25] Reichling C., Nobile L., Pezzullo M., Navez J., Bachir N., D’Haene N., Maris C., Musala C., Fernandez Y., Viesca M. (2020). Non-occlusive Mesenteric Ischemia as a Fatal Complication in Acute Pancreatitis: A Case Series. Dig. Dis. Sci..

[26] Edwards M.S., Cherr G.S., Craven T.E., Olsen A.W., Plonk G.W., Geary R.L., Ligush J.L., Hansen K.J. (2003). Acute occlusive mesenteric ischemia: Surgical management and outcomes. Ann. Vasc. Surg..

[27] Salsano G., Salsano A., Sportelli E., Petrocelli F., Dahmane M., Spinella G., Pane B., Mambrini S., Palombo D., Santini F. (2018). What is the Best Revascularization Strategy for Acute Occlusive Arterial Mesenteric Ischemia: Systematic Review and Meta-analysis. Cardiovasc. Interv. Radiol..

[28] Orsi F., Zhang L., Arnone P., Orgera G., Bonomo G., Vigna P.D., Monfardini L., Zhou K., Chen W., Wang Z. (2010). High-intensity focused ultrasound ablation: Effective and safe therapy for solid tumors in difficult locations. Am. J. Roentgenol..

[29] Ligresti D., Kuo Y.T., Baraldo S., Chavan R., Keane M.G., Seleem S., Seo D.W. (2019). EUS anatomy of the pancreatobiliary system in a swine model: The WISE experience. Endosc. Ultrasound.

[30] Ferrer J., Scott W.E., Weegman B.P., Suszynski T.M., Sutherland D.E., Hering B.J., Papas K.K. (2008). Pig pancreas anatomy: Implications for pancreas procurement, preservation, and islet isolation. Transplantation.

[31] Khokhlova T.D., Hwang J.H. (2016). HIFU for Palliative Treatment of Pancreatic Cancer. Adv. Exp. Med. Biol..

[32] Hwang J.H., Wang Y.N., Warren C., Upton M.P., Starr F., Zhou Y., Mitchell S.B. (2009). Preclinical in vivo evaluation of an extracorporeal HIFU device for ablation of pancreatic tumors. Ultrasound Med. Biol..

[33] Huang G., Ye X., Yang X., Zheng A., Li W., Wang J., Han X., Wei Z., Meng M., Ni Y. (2019). Experimental study in vivo ablation of swine pancreas using high-intensity focused ultrasound. J. Cancer Res. Ther..

[34] Xie B., Li Y.Y., Jia L., Nie Y.Q., Du H., Jiang S.M. (2010). Experimental ablation of the pancreas with high intensity focused ultrasound (HIFU) in a porcine model. Int. J. Med. Sci..

[35] Chang W., Lee J.Y., Lee J.H., Bae J.S., Cho Y.J., Kang K.J., Son K., Chung Y.R., Lee K.B., Han J.K. (2018). A portable high-intensity focused ultrasound system for the pancreas with 3D electronic steering: A preclinical study in a swine model. Ultrasonography.

[36] Sebeke L.C., Rademann P., Maul A.C., Schubert-Quecke C., Annecke T., Yeo S.Y., Castillo-Gomez J.D., Schmidt P., Grull H., Heijman E. (2020). Feasibility study of MR-guided pancreas ablation using high-intensity focused ultrasound in a healthy swine model. Int. J. Hyperth..

[37] Sanchez M., Barrere V., Treilleux I., Chopin N., Melodelima D. (2021). Development of a noninvasive HIFU treatment for breast adenocarcinomas using a toroidal transducer based on preliminary attenuation measurements. Ultrasonics.

[38] Cilleros C., Dupre A., Vincenot J., Melodelima D. (2021). Development of a Simple In Vitro Artery Model and an Evaluation of the Impact of Pulsed Flow on High-Intensity Focused Ultrasound Ablation. IRBM.

[39] Battais A., Barrere V., N’Djin W.A., Dupre A., Rivoire M., Melodelima D. (2020). Fast and Selective Ablation of Liver Tumors by High-Intensity Focused Ultrasound Using a Toroidal Transducer Guided by Ultrasound Imaging: The Results of Animal Experiments. Ultrasound Med. Biol..

[40] Caloone J., Huissoud C., Vincenot J., Kocot A., Dehay C., Chapelon J.Y., Rudigoz R.C., Melodelima D. (2015). High-intensity focused ultrasound applied to the placenta using a toroidal transducer: A preliminary ex-vivo study. Ultrasound Obstet. Gynecol..

[41] N’Djin W.A., Melodelima D., Schenone F., Rivoire M., Chapelon J.Y. (2011). Assisted hepatic resection using a toroidal HIFU device: An in vivo comparative study in pig. Med. Phys..

[42] Klaiber U., Schnaidt E.S., Hinz U., Gaida M.M., Heger U., Hank T., Strobel O., Neoptolemos J.P., Mihaljevic A.L., Buchler M.W. (2021). Prognostic Factors of Survival After Neoadjuvant Treatment and Resection for Initially Unresectable Pancreatic Cancer. Ann. Surg..

[43] Michelakos T., Pergolini I., Castillo C.F., Honselmann K.C., Cai L., Deshpande V., Wo J.Y., Ryan D.P., Allen J.N., Blaszkowsky L.S. (2019). Predictors of Resectability and Survival in Patients With Borderline and Locally Advanced Pancreatic Cancer who Underwent Neoadjuvant Treatment With FOLFIRINOX. Ann. Surg..

[44] Elias D., Baton O., Sideris L., Lasser P., Pocard M. (2004). Necrotizing pancreatitis after radiofrequency destruction of pancreatic tumours. Eur. J. Surg. Oncol..

[45] Girelli R., Frigerio I., Giardino A., Regi P., Gobbo S., Malleo G., Salvia R., Bassi C. (2013). Results of 100 pancreatic radiofrequency ablations in the context of a multimodal strategy for stage III ductal adenocarcinoma. Langenbeck Arch. Surg..

[46] Fegrachi S., Walma M.S., de Vries J.J.J., van Santvoort H.C., Besselink M.G., von Asmuth E.G., van Leeuwen M.S., Rinkes I.H.B., Bruijnen R.C., de Hingh I.H. (2019). Safety of radiofrequency ablation in patients with locally advanced, unresectable pancreatic cancer: A phase II study. Eur. J. Surg. Oncol..

[47] Ruarus A.H., Vroomen L., Geboers B., van Veldhuisen E., Puijk R.S., Nieuwenhuizen S., Besselink M.G., Zonderhuis B.M., Kazemier G., de Gruijl T.D. (2020). Percutaneous Irreversible Electroporation in Locally Advanced and Recurrent Pancreatic Cancer (PANFIRE-2): A Multicenter, Prospective, Single-Arm, Phase II Study. Radiology.

[48] He C., Wang J., Sun S., Zhang Y., Lin X., Lao X., Cui B., Li S. (2019). Irreversible electroporation versus radiotherapy after induction chemotherapy on survival in patients with locally advanced pancreatic cancer: A propensity score analysis. BMC Cancer.

[49] Stanislavova N., Karamanliev M., Ivanov T., Yotsov T., Zhou K., Dimitrov D. (2021). Is high-intensity focused ultrasound (HIFU) an option for neoadjuvant therapy for borderline resectable pancreatic cancer patients?—A systematic review. Int. J. Hyperth..

[50] Marinova M., Huxold H.C., Henseler J., Mucke M., Conrad R., Rolke R., Ahmadzadehfar H., Rauch M., Fimmers R., Luechters G. (2019). Clinical Effectiveness and Potential Survival Benefit of US-Guided High-Intensity Focused Ultrasound Therapy in Patients with Advanced-Stage Pancreatic Cancer. Ultraschall Med..

[51] Zhu B., Li J., Diao L., Ma K., Fan Y., Yang W. (2019). High-intensity focused ultrasound ablation for advanced pancreatic cancer. J. Cancer Res. Ther..

[52] Delpero J.R., Bachellier P., Regenet N., le Treut Y.P., Paye F., Carrere N., Sauvanet A., Autret A., Turrini O., Monges-Ranchin G. (2014). Pancreaticoduodenectomy for pancreatic ductal adenocarcinoma: A French multicentre prospective evaluation of resection margins in 150 evaluable specimens. HPB.

[53] N’Djin W.A., Chapelon J.Y., Melodelima D. (2015). An Ultrasound Image-Based Dynamic Fusion Modeling Method for Predicting the Quantitative Impact of In Vivo Liver Motion on Intraoperative HIFU Therapies: Investigations in a Porcine Model. PLoS ONE.

[54] Vincenot J., Melodelima D., Chavrier F., Vignot A., Kocot A., Chapelon J.Y. (2013). Electronic beam steering used with a toroidal HIFU transducer substantially increases the coagulated volume. Ultrasound Med. Biol..

